# Uptake, Elimination and Effects of Cosmetic Microbeads on the Freshwater Gastropod *Biomphalaria glabrata*

**DOI:** 10.3390/toxics10020087

**Published:** 2022-02-14

**Authors:** Ying Wang, Alice Baynes, Kofi O. Renner, Mingxing Zhang, Mark D. Scrimshaw, Edwin J. Routledge

**Affiliations:** 1 Key Laboratory for Ecological Environment in Coastal Areas, Ministry of Ecology and Environment, National Marine Environmental Monitoring Center, 42 Linghe Street, Dalian 116023, China; mxzhang@nmemc.org.cn; 2 Institute of Environment, Health and Societies, Brunel University London, Uxbridge UB8 3PH, UK; Alice.Baynes@brunel.ac.uk (A.B.); Kofi.Renner@environment-agency.gov.uk (K.O.R.); Mark.Scrimshaw@brunel.ac.uk (M.D.S.)

**Keywords:** ingestion, aggregation, toxicity, microbeads, snail

## Abstract

The presence of plastic cosmetic microbeads in the environment due to their extensive use in society and inevitable dispersal into wastewater is concerning. Therefore, it is vital to understand the processes of microplastic uptake and elimination by aquatic organisms, and to further assess their potential to cause harmful effects and wider impacts. We therefore investigated the short-term (48-h) and long-term (21-d) uptake, elimination, and effects of exposure to polyethylene microbeads (a mixture of fragments and spheres extracted from commercially available facial scrubs) on the freshwater snail, *Biomphalaria glabrata*. We found fast uptake in the short-term (75 μg/g/h) and the long-term (6.94 μg/g/h) in *B. glabrata* exposed to 800 particles/200-mL and 80 particles/200-mL, respectively. Irregular fragments were more easily ingested and egested compared to spheres (ANOVA, *p* < 0.05) in both 48-h and 21-d exposures. The mean size of the fragments in *B. glabrata* tissues (413 ± 16 μm) after 48-h exposure was significantly larger than that of the standard sample (369 ± 26 μm) (ANOVA, *F*_3,20_ = 3.339, *p* = 0.033), suggesting that aggregation in the gut may occur. Floating feces containing microbeads were observed in the long-term exposure, which could alter the fate, behavior, and bioavailability of egested microbeads. No significant effects on survival and growth were shown within 48-h or 21-d exposure periods. Thus, further studies on the specific features of microplastics (e.g., their shape and size) influencing uptake and elimination, as well as toxic molecular mechanisms, should be explored in future ecotoxicological studies.

## 1. Introduction

Microplastic pollution is now prevalent in all aquatic habitats including rivers, bays, estuaries, and the deep sea around the world [1,2,3,4,5]. Here, we consider a subset of microplastics (MPs) known as ‘microbeads’, which are fragments or beads of plastic ranging from roughly 5 μm to 1 mm in size that do not readily biodegrade in nature [6]. Microbeads are intentionally manufactured and incorporated into various personal care products (including facial scrubs and body wash) as exfoliants, as well as industrial and household cleaning products as abrasives [7,8]. Microbeads incorporated in personal care products are typically washed down the drain during normal use. As current wastewater treatment plants (WWTPs) technologies cannot remove microbeads due to their small size, they are consequently emitted into the environment, and are detected in both final effluent and sewage sludge produced from WWTPs. Although often barely visible to the naked eye, the accumulation of microplastics in the aquatic environment is an issue of emerging concern. Indeed, it was conservatively estimated that, every day, 8 billion microbeads enter into aquatic habitats within the United States alone [6].

Fields studies have shown that MPs of different shapes are bioavailable to a wide range of aquatic species [1] from invertebrates [9] to fish [10] and whales [11], where they are mistaken as food. Microbeads (fragments and beads) and fibers (mainly from synthetic clothes released by washing) are now the two most commonly reported shapes [12,13,14]. Indeed, microbeads, as with all MPs, have been reported in wild Japanese anchovy as both fragments (86.0%) and beads (7.3%), similar to those found in facial cleansers [15].

Of the potential negative impacts of microplastic ingestion on aquatic organisms, physical injury of the gastrointestinal tracts and impaired nourishment resulting in adverse effects, such as decreased fecundity, are reported [16,17]. However, such reported effects are often associated with short-term exposures using very high concentrations of MPs. To better understand the impact of MPs on aquatic ecosystems and to generate data that is useful to regulators, it is important to conduct “environmentally relevant” exposures in ecotoxicology research [18,19]. More recently, environmentally relevant microplastic sources and forms, including polyethylene microbeads, are being investigated instead of fluorescently labelled or non-labelled research-grade plastic microspheres [20,21]. This is important, since morphology (e.g., fragments, spheres, and fibers) influences the ingestion rate of MPs by grass shrimp (*Palaemonetes pugio*), retention time in goldfish guts (*Carassius auratus*), and toxic effects on the water flea (*Ceriodaphnia dubia*) [22,23,24]. In mollusks, no morphological, life-history parameter alterations have been reported for the freshwater gastropod, *Potampoyrgus antipodarum*, which has been exposed to several environmentally relevant non-buoyant polymers [25]. Similarly, exposure to irregular polystyrene produced no significant effects on survival, reproduction, energy reserves, or oxidative stress of the gastropod, *Lymnaea stagnalis* [26].

The importance of using environmentally relevant concentrations in ecotoxicology research has also been emphasized. Indeed, the experimental conditions used by Sussarellu et al. published in PNAS (2 and 6 μm in diameter; 0.023 mg/L) [27] may provide such insights, but others use concentrations that are orders of magnitude higher than those reported in field studies [18]. Therefore, ecotoxicological experiments at environmentally relevant concentrations are necessary to determine the toxic effects of ingested MPs in different species. However, there is limited knowledge about the ingestion and subsequent adverse effects of real-world “environmentally relevant” MPs used in society, particularly microbeads from personal care products, on freshwater biota.

The tropical freshwater snail, *Biomphalaria glabrata*, was selected as the test species due to its well-documented physiology and animal husbandry, and because it represents a large group of freshwater gastropods in terms of its feeding habits [28,29]. The aim of this study was to examine (i) the ingestion and elimination of polyethylene microbeads with different shapes (fragments and spheres) extracted from facial scrubs, and (ii) the effects of the ingested microbeads on growth and survival of *B. glabrata*. Both the acute (48-h) and chronic (21-d) exposure experiments were designed using microplastic concentrations previously reported for surface waters in the southern North Sea (maximum 1770 particles/L) [30] and Swedish coastal waters (maximum 102 particles/L) [31]. The microbeads recovered from different media (tissue, feces, water) were analyzed to investigate their size distribution and shape. This present study would be useful to further assess ecological risks of microbeads in aquatic environments.

## 2. Materials and Methods

### 2.1. Extraction of Microbeads from Facial Scrub

**Figure 1:**
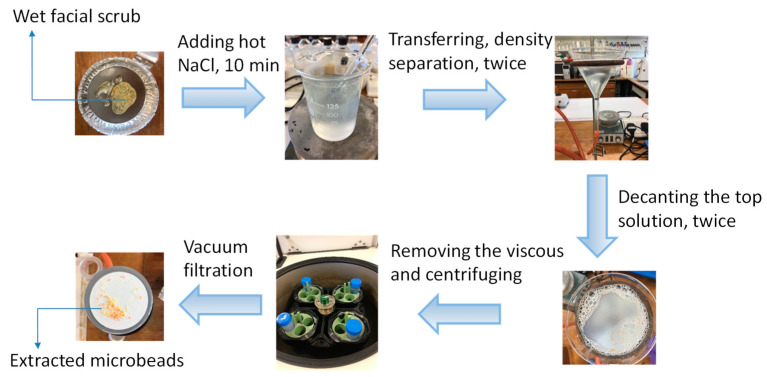
Schematic diagram of the whole process to extract microbeads from facial scrub for use in the exposure studies. More details are given in Section 2.1.

The extraction process of microbeads from facial scrub (Clean and Clear Morning Energy Skin Energizing Daily) shown in Figure 1 was based on density separation using sodium chloride. Firstly, 0.5 g (wet weight) of the product was dissolved in 1-mL of warm Milli-Q ultrapure water (50–60 °C) and stirred using a glass rod. Then, 50-ml of sodium chloride (140 g/L) at a temperature of 50–60 °C was then added to the dispersion solution in a 200-mL glass beaker and mixed for 10 min. The resulting solution was transferred into a glass funnel. Afterwards, the floating particles on the surface were collected by decanting the top solution (~15-mL) into a clean glass beaker. The density separation process was repeated twice, after which ~30-mL of resulting solution was then reduced into 15-mL by removing the viscous material at the bottom of the beaker using a pipette. The residual 15-mL of solution was transferred to a 50-mL of centrifuge tube, to which 35-mL of hot sodium chloride solution (50–60 °C) was added. The centrifugation process was carried out at 1700× *g* for 4 min, after which the top layer of the solution containing MPs (~10-mL) was decanted into a clean 50-mL beaker. Finally, the 10-mL of mixture solution was vacuum filtered through a glass fiber filter (Whatman, GF/C, pore size 1.2 μm). The filters and retained particles were dried at 50 °C in an oven for 5 h. Microbeads on the filters were gently collected in glass petri dishes (Figure S1A) and kept in the desiccator before using.

### 2.2. Quantification of Microbeads

The relationship between the number of microbeads (expressed as particles/mL) and their concentration (expressed as mg/L) is necessary to calculate in order to compare our measures to other reported environmental concentration (particles/m^3^) and exposure concentration in bioassays (mg/L) [24]. To determine the numbers of microbeads extracted from the facial scrub, we weighed 2 mg of them as a “standard sample” (Figure S1B) since the number (~160 particles ± 20) was appropriate for statistical analysis for the different shapes under microscope. Images were obtained for various samples including the “standard sample”, tissue, feces, and the exposure medium samples under stereomicroscope (Olympus SZX12, Tokyo, Japan). Counting and size measurements for fragments and spheres (*n* = 6 replicates) were performed using Image J (Version 1.51k) [24]. The Feret diameter of all the particles extracted was calculated using sizing and circularity parameters.

### 2.3. Identification of Microbeads

The polymer composition of microbeads was identified using Attenuated Total Reflection Fourier-Transform Infrared (ATR-FTIR) spectroscopy (PerkinElmer RX1, Beaconsfield, UK). The spectra scans were carried out from a wavenumber range of 4000 cm^−1^ to 700 cm^−1^ at a spectral resolution of 4 cm^−1^.

### 2.4. Digestion Method

We used the following formula modified from Karami et al. [32] to calculate the digestion efficiency (%) (*n* = 6 per treatment):

$$\mathrm{Digestion}\;\mathrm{efficiency}\;\left(\%\right)=\frac{W_{i}-\left(W_{a}-W_{b}-W_{c}\right)}{W_{i}}\times 100$$

where *W*_i_ is initial dry weight of biological materials, *W*_a_ is weight of dry filter membrane after filtration, *W*_b_ is weight of dry filter membrane before filtration, and *W*_c_ is the weight change of filter membrane by chemical treatment. Digestion efficiency tests were performed in two independent experiments. The detailed stepwise approach employed in this study was described below:

The dissected soft tissues of the whole body from four adult individuals (body length, 7.6 ± 1.1 mm) were transferred into 50-mL glass beakers holding 40-mL of 10% potassium hydroxide (KOH) per gram wet weight (*n* = 6 replicates) at room temperature for four days. Beakers were shaken by hand every day to facilitate the contact of the tissues with the digestion reagent. Digestates were filtered through a filter paper (Whatman No. 540, pore size 8 μm) under vacuum. Before and after filtration, the filter membranes were maintained at 50 °C for 5-h and then weighed on a scale with 0.1 mg precision.

### 2.5. Test Organism

The freshwater gastropod *B. glabrata* (BB02 strain) stock (originally sourced from The Natural History Museum, London, UK) have been cultured at Brunel University London’s aquatic facility since 2010. Snails were maintained in a flow-through system in glass aquaria supplied with de-chlorinated tap water, and were fed ad libitum every other day with TetraMin fish flakes (~10% of their body mass). A light regime of 16: 8 L: D, including 20 min dawn/dusk transition periods, was maintained throughout the experiment. Tank water conditions were monitored throughout the study; temperature (27 ± 1 °C), pH (8 ± 0.21), dissolved oxygen (6 ± 2 mg/L) and ammonia (0 mg/L), nitrate (0 mg/L), and nitrite (<40 mg/L) [25].

### 2.6. Experimental Design

Experiments were designed and conducted to study the uptake, elimination, and effects of exposure to MPs derived from facial scrub on *B. glabrata* (Table S1). In Exp. I(a) and Exp. I(b), the accumulation and effect of microbeads on survival and growth were examined during a short-term (48-h) exposure. In Exp. II(a) and Exp. II(b), the accumulation and effects of MPs on survival and growth were examined during a long-term (21-d) exposure.

### 2.7. Short-Term (48-h) Exposure

A 48-h exposure was conducted to examine the short-term ingestion, egestion (Exp. I(a)) and changes in body length and weight (Exp. I(b)) of microbeads on adult *B. glabrata*. Both yellow beads and white fragments were included in the two experiments. Briefly, 10-mg of MPs were weighed and added to a glass petri dish containing 200-mL dechlorinated tap water, culture medium of *B. glabrata*. Tween-20 surfactant (0.0025%, *v/v*) was used to disperse the microplastic solution, which was mixed completely using a glass rod [33]. Food was not provided for the short-term exposure. A single dose of plastic particles was adopted to achieve a nominal final concentration of 5 mg/L (equivalent to four particles/mL). In total, 60 3-month-old adult snails (*n* = 5 per replicate) were exposed in 200-mL glass petri dishes per treatment (exposed and unexposed). The light and temperature conditions were the same as those specified above (test organism).

After 48-h of exposure, the mortality rates of B. glabrata were calculated. All individuals were weighed (g) and shell diameters were measured (mm) prior to tissue sampling. The entire soft tissue was washed three times with milli-Q water to remove any MPs present on the outside surface, and each replicate of the two treatment groups was then split. In total, four individuals per replicate were pooled and transferred to 50-mL glass beakers for digestion to quantify MPs in B. glabrata (body burden). One individual/replicate was fixed with Bouin’s solution (Sigma-Aldrich, Dorset, UK) for histopathological assessment (data not shown). In Exp. I(a), we also quantified the microbeads remaining in the aqueous exposure medium after the exposure by filtering the solution and counting under stereomicroscope. Feces from each replicate group of snails were also collected and pooled, inspected under stereomicroscope, and digested by KOH to quantify the egested microbeads.

### 2.8. Long-Term Exposure (21 d)

A 21-d exposure was conducted to investigate the accumulation (Exp. II(a)) and chronic toxic effects (Exp. II(b)) of microbeads on 2-month-old *B. glabrata*. There were some experimental design differences between the long-term and short-term exposure. Firstly, in the long-term exposure, 1-mg of MPs was weighed for each replicate group with the final concentration of 0.5-mg/L (equivalent to 0.4 particle/mL). Secondly, the exposure medium containing MPs was totally exchanged every other day followed by the addition of food supply (~10% body weight). Moreover, feces of each group were collected, pooled, inspected, and quantified on day 2, 4, 6, 8, 16, and 21 to investigate the characteristics of the egested microbeads. Lastly, four individuals per replicate were pooled and transferred to 50-mL glass beakers for digestion to quantify MPs in *B. glabrata* (body burden).

### 2.9. Statistics

Statistical analysis was conducted using SPSS 16.0 (SPSS, Chicago, IL, USA). Analysis of variance (ANOVA) with Tukey’s post hoc test or paired *t*-test was used to compare the various treatments, and *p* values < 0.05 were considered significant. Data are reported as mean ± standard deviation.

## 3. Results and Discussions

### 3.1. Shape, Size and Chemical Composition of Microbeads in Facial Scrub

The extracted microbeads from the commercial facial scrub contained white fragments and orange/yellow spheres which were visible to the naked eye (Figure S1A). Orange microspheres were excluded in the bioassays since they were too large (around 1 mm diameter) to be ingested by snails in our preliminary trials (Figure S1B). The “standard samples” prepared from the facial scrub contained microbeads with diameters ranging from 83 μm to 789 μm in size (mean 346 ± 88 μm; Figure S2). The proportion of white fragments exceeded the yellow spheres in “standard samples” by a factor of 2.4 ± 0.8 (*n* = 6). FT-IR analysis results confirmed that the chemical composition of microbeads was polyethylene (Figure S3), a common polymer. The mass concentration of 5 mg/L of microbeads was equivalent to 0.4 ± 0.05 particles/mL (*n* = 6), based on counting under the stereomicroscope.

The average diameter (346 ± 88 μm) of test facial scrub particles in this study is higher than that reported for nine different brand facial scrubs (85–186 μm) [8]. This may be a result of differences in the brands and the counting method [34]. Although ImageJ was used in both studies, manual measurements are preferable as they are more accurate than automated measurements that are reliant on the judgment of microbeads using software. The shapes of “microbeads” have been found to be irregular fragments or regular spheres, and the irregular fragments were more abundant compared to the spherical beads in this study, which is consistent with previous studies [8,15].

### 3.2. Uptake and Elimination of Microbeads in Short-Term and Long-Term Assays

**Figure 2:**
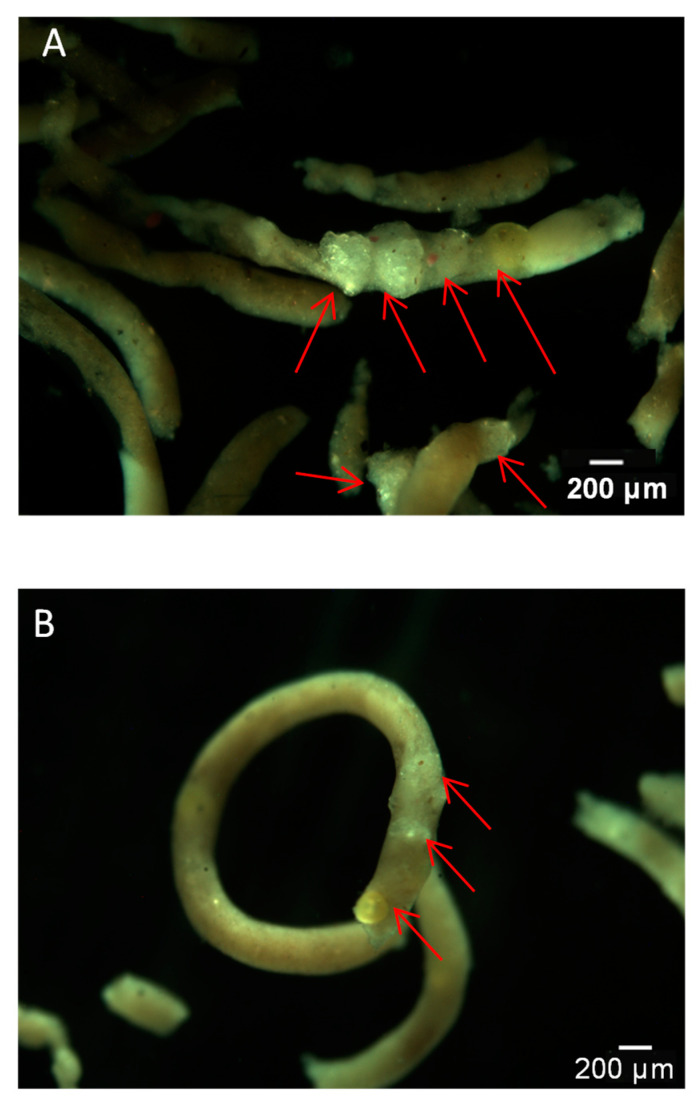
Feces of *B. glabrata* with fragments and spheres upon exposures to 4 particles/mL polyethylene microbeads for 48 h (**A**) and 0.4 particles/mL polyethylene microbeads for 21 d (**B**).

All snails survived in both Exp. I(a) and Exp. II(a). The first-hand evidence of polyethylene microbead ingestion by snails in short-term (48-h) and long-term (21-d) exposure assays were obtained microscopically by inspecting the feces (Figure 2). MPs, including both spheres and fragments, were detected in both tissues and feces after the short-time and long-term exposures. No MPs were found in control snails (Figure S4). No mortality was observed in both the short-term and long-term experiments.

#### 3.2.1. Short-Term Exposure Assay

**Figure 3:**
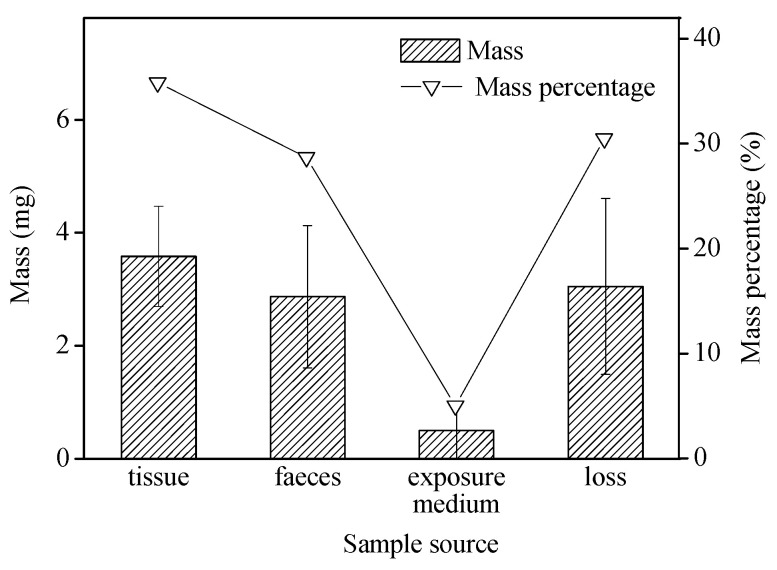
Microbead mass (mean ± standard deviation; *n* = 6) and its percentage in different media over 48-h exposure.

In our study, *B. glabrata* exposed to 1 mg microbeads per 200-mL for 48 h displayed fast uptake (75 μg/g/h), which was slightly higher than observed with krill (*Euphausia superba*) exposed to a 20% plastic diet for 48 h (22 μg/g/h) [35]. The wet weight of internalized microbeads (microplastic particles left in the tissue, *n* = 6; shown in Figure 3) for these snails over 48-h exposure was on average 3.6 ± 0.9 mg/g (equivalent to 54.5 ± 26.3 particles/individual), accounting for 35.8 ± 8.9% of the total microbeads used in this treatment. In comparison, 2.9 ± 1.2 mg of the microbeads were egested in the feces (*n* = 6), representing 28.7 ± 12.7% of the total microbeads offered in this treatment (Figure 3). Particles recovered from the exposure media only accounted for approximately 5% of the total, suggesting rapid ingestion and egestion of microbeads by snails over 48-h exposure. When measured, approximately 30% of the microbeads were unaccounted for, i.e., not found in the snails’ tissues, feces, or in the exposure medium. The microbead loss may be due to adsorption to the vessel walls, and during the filtration process or via transfer from different vessels. Polyethylene microbeads, added at the start of the exposure, were seen floating on the surface of the exposure media due to their lower specific gravity.

**Figure 4:**
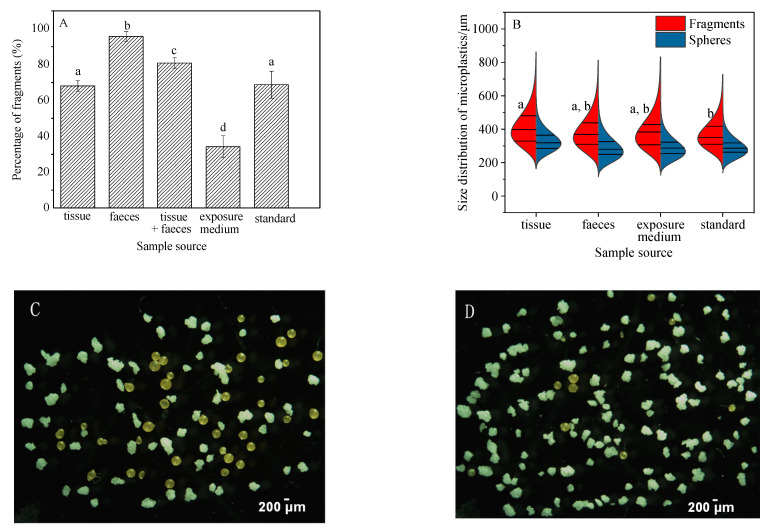
Shape (**A**) and size (**B**) distribution of microplastics in different media (tissue, feces, and exposure medium) over 48-h exposure. Typical extracted microplastics in tissue (**C**) and feces (**D**) exposure medium samples are shown. Straight lines in Figure 4B show medians and interquartile. Differences between groups were analyzed for significance using Tukey’s multiple comparison test. Different letters above columns in the histogram and one side in the violin plot indicate significant differences (*p* < 0.05).

Significant variations in the percentage of fragments present in samples of feces, tissue + feces, exposure medium, and standard were seen (Figure 4A, ANOVA, (*F*_(4,25)_ = 127.927, *p* < 0.05)). Fragments detected in feces accounted for 95.7% ± 2.7% of the total particles recovered, which was higher than that in the original “standard sample” (68.8 ± 7.5%) (Figure 4, Figure S1B). The high percentage of fragments in the feces suggests that they were more easily ingested and egested compared to the spheres. In other words, ingested spheres may be retained longer in the snails. The higher proportion of fragments ingested and egested in the feces also resulted in a lower percentage (34.2% ± 6.1%) of free fragments in the exposure medium at the end of the experiment (Figure 4A). Similar results were also obtained for grass shrimp (*Palaemonetes pugio*), where the number of fragments ingested by shrimp were significantly higher than spheres [22]. Plastic fragments were also isolated as the dominant shape from three different species of edible snails belonging to the genus Helix in the terrestrial ecosystem [36].

Overall, the gastropod *B. glabrata* demonstrated no significant variation in the size of the fragments recovered from feces, exposure media and the original standard. Neither were differences observed in the size distribution of spheres recovered from tissue, feces, exposure media, and the original standard. Interestingly, however, the average size of fragments accumulated in snail tissue was 413 ± 16 μm, which is significantly higher than that in the standard sample (369 ± 26 μm) (ANOVA, *F*_(3,20)_ = 3.339, *p* = 0.033) (Figure 4B). This may be a result of aggregation behavior of internalized MPs in *B. glabrata.* Microplastic aggregation is a process whereby two or more microplastic particles fuse with each other when they collide [37]. A large number of studies have investigated the homoaggregation and hetrobehavior of MPs [38]. For instance, MPs could interact with micro- and macro-algae and aquatic plants to further form aggregates [39]; aggregation of polyethylene particles has been reported to occur in the gut of planktivorous fish [15]. Interactions between MPs and gut biota may also influence how MPs are repackaged into feces [40]. Indeed, assortative processes during ingestion and digestion are likely to have resulted in the relatively larger size of polyethylene particles in the tissues of *B. glabrata* compared to the standard microplastic sample in this study. It could be speculated that the polyethylene fragments interacted with the residual flake food and/or bacteria in the digestive tract of *B. glabrata* in this study to form an eco-corona [40] that encouraged further aggregation. Alternatively, it may be attributed to the active selection of larger particles of *B. glabrata*, as they were not being fed during the 48-h exposure.

#### 3.2.2. Long-Term Exposure Assay

**Figure 5:**
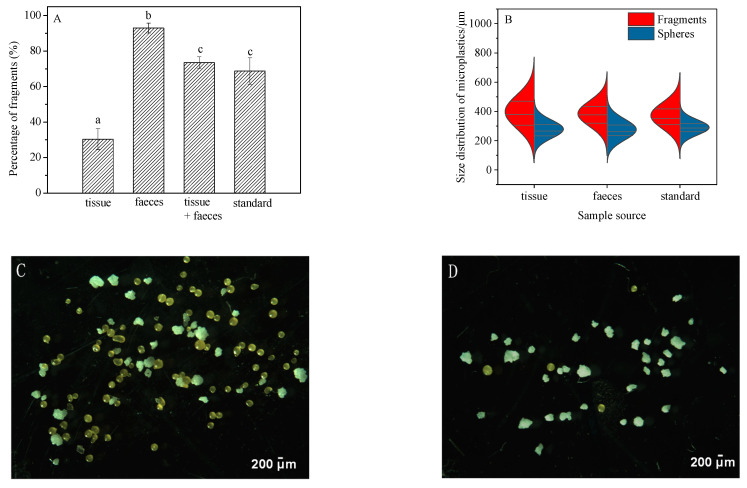
Shape (**A**) and size (**B**) distribution of microplastics in different media during the 21-d exposure. Typical photographs of typical extracted microbeads in tissue (**C**) and feces (**D**) over the 21-d exposure are shown. Differences between groups were analyzed for significance using Tukey’s multiple comparison test. Different letters above columns in subgraph (**A**) indicate significant differences (ANOVA, *p* < 0.05). Straight lines in subgraph (**B**) show medians and interquartile. No significant differences in the size of fragments and spheres exist in Figure 5B using Tukey’s multiple comparison test (ANOVA, *p* > 0.05).

**Figure 6:**
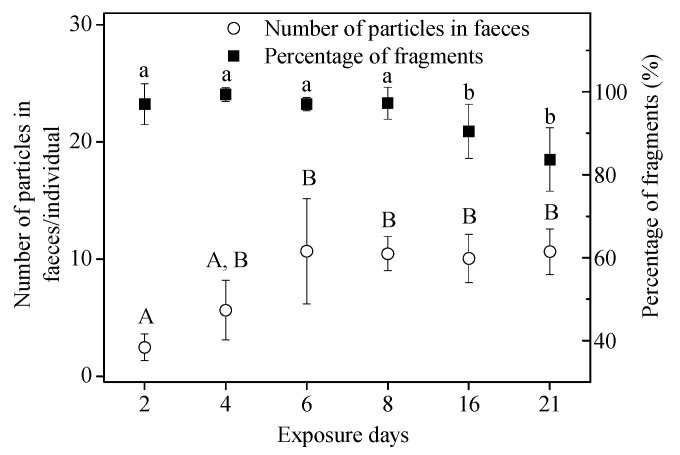
Number of particles in feces collected at Day 2, 4, 6, 8, 16, and 21 during the 21-d exposure. Different letters above columns indicate significant differences (ANOVA, *p* < 0.05).

In the 21-d, long-term assay, no microbeads were detected in the controls. The cumulative number of MPs in tissues of snails for microbead exposure was 22.3 ± 4.5 particles/individual (3.5 ± 1.0 mg/g WW). For the percentage of fragments present in samples of feces, tissue + feces, and standard, similar results to those in the short-term assay were obtained (Figure 5A, ANOVA, (*F*_(__3,2__3)_ = 146.928, *p* < 0.05)). Fragments detected in feces accounted for 92.9% ± 2.8% of the total particles recovered, which was significantly higher than that in the original “standard sample” (68.8 ± 7.5%) (Figure 5A). At the same time, there were no significant differences in the size of fragments (ANOVA, *F*_2,15_ = 2.854, *p* = 0.089) and spheres (ANOVA, *F*_2,15_ = 0.918, *p* = 0.421) recovered from the three groups (tissue, feces, and standard sample) over the 21-d exposure (Figure 5B). The high percentage of fragments in the feces also suggests that they were more easily ingested and egested compared to the spheres for the long-term assay, and that ingested spheres may be retained longer in the snails. The number of particles in the feces increased over time from 2.5 ± 1.1 particles/individual (Day 2) to 10.7 ± 4.5 particles/individual (Day 6), and then remained stable (Figure 6). The percentage of fragments in feces exceeded 80% (Figure 6); significantly higher than in the standard sample (68.8 ± 7.5%) (ANOVA, *F*_7,40_ = 111.007, *p* < 0.05).

The uptake rate of MPs (80 particles/200-mL) in young snails in the long-term (21 day) assay was 6.94 μg/g/h. This was one order of magnitude lower than the uptake rate in the short-term (48 h) assay (75 μg/g/h), and is likely to be a consequence of the lower initial microplastic particle concentration in the chronic study. The percentage of fragments in feces over the short-term (48-h) and the long-term (21-d) were 95.7 ± 2.8% and 83.6 ± 7.6%, respectively. This suggests that the fragments are more easily ingested and egested compared to spheres for both short-time and long-time microplastic exposures. The typical microscopic photographs for the low ratio (fragments/spheres) in tissue and the high ratio (fragments/spheres) in feces are also shown in Figure 5C,D, respectively. Our result also revealed no significant differences in the size of fragments and spheres measured over the 21-d exposure in the three media. The average size of fragments accumulated in snail tissue over long-term (21 d) exposure was 400 ± 27 μm, significantly lower than that (413 ± 16 μm) over short-term (48 h) exposure (paired *t*-test, *t* = 61, *p* < 0.05). It is possible that existing differences in size compared with the short-term assay might be attributed to the relatively lower microplastic exposure concentration, which resulted in no aggregation.

An interesting finding in the long-term exposure was the occurrence of floating feces containing microbeads (Figure 2B), instead of the usual situation where feces remain at the vessel bottom. In contrast, in the short-term assay, feces containing microbeads were found in the vessel bottom (Figure 2A). This might be attributed to the aging process of exposed MPs over the longer exposure, resulting in more removal of additives and fillers and the formation of biofilm [21]. Young snails probably ingested aged MPs multiple times, although we replenished the water every other day. These changes could make the microplastic less dense. Different types of MPs including low density polyethylene, medium density nylon, and high-density polyethylene terephthalate can alter the feeding selectivity and fecal density in the copepod [41]. In agreement with previous findings in other biological systems, feces (as a vector for transport of MPs) are also a source of food for aquatic organisms, and contribute to the vertical flux of particulate matter and their access into the food web [42,43]. The process of ingestion and egestion by organisms could change the density of MPs and their fate in the aquatic environment and further affect their bioavailability to aquatic organisms such as detritus feeders and zooplankton [40,44]. For example, research results showed that MPs, encapsulated within fecal pellets of the copepod *Centropages typicus*, could be transferred to copepod *Calanus helgolandicus* via coprophagy [42]. This could also increase their potential to enhance the transport of other pollutants absorbing on the MPs [37,45].

### 3.3. In Vivo Effects

The survival of snails was not affected by the 48-h/21-day exposure to MPs in Exp. I(b) and Exp. II(b). Additionally, there were no significant differences in body length increment (ANOVA, *F*_1,10_ = 0.551, *p* = 0.475) and body weight increment (ANOVA, *F*_1,10_ = 0.611, *p* = 0.453) between the microplastic treatment groups and the control groups (Figure 6). Therefore, there were no significant growth effects of microbeads (polyethylene fragments and spheres) on *B. glabrata* over short-term (48 h) and long-term (21 d) exposures.

The limited impacts on aquatic organisms upon exposure and ingestion of microplastics are in agreement with previous findings in aquatic invertebrates, unlike the detrimental physical effects of large plastic items. For instance, limited acute toxicity (no mortality or dose dependent weight loss) of ingested MPs (polyethylene microspheres, 27–32 μm) occurred in exposed krill (*Euphausia superba*) during a 10-day assay [35]. Polyethylene microspheres (10–45 μm) had a small effect on larval growth and no significant effect on survival of sea urchin, *Tripneustes gratilla*, even at a concentration far exceeding those recorded in the marine environment [46]. Similarly, adult mud snails *Potamopyrgus antipodarum* showed no morphological changes including shell size and weight after exposure to a large range of common and environmentally relevant microplastic particles such as polyamide and polyethylene (4.64–602 μm) [25]. An exposure to irregular 5–90 μm spherical polystyrene MPs had no effect on survival, reproduction, energy reserves, and oxidative stress of the freshwater gastropod *Lymnaea stagnalis* [26].

Importantly, adverse effects on feeding, function, and fecundity of marine and freshwater invertebrates exposed to polyethylene MPs has been reported in previous studies [17,24]. This might be attributed to their small size (less than 20 μm) and relatively high exposure concentrations. Effects might be highly dependent on such environmentally relevant factors including the abundance of MPs, chemical composition of the polymer itself, and the size and shape of MPs detected in the real environment [25,47]. Therefore, it is important to further investigate the population-level effects with the consideration of their environmentally relevant factors mentioned above.

## 4. Conclusions

Overall, this study reported the uptake and elimination of polyethylene microbeads from facial scrub on the freshwater gastropod *B. glabrata* over 48 h and 21 days, and their toxic effects of ingested MPs. Our results demonstrated that fragments were more easily ingested and egested compared with spheres, and their aggregation might have occurred in *B. glabrata* in the short-term bioassay. Floating feces containing microbeads were observed in the long-term bioassay, which might change MP fate and further their bioavailability. No significant impacts on survival or growth were observed in *B. glabrata* within 48-h or 21-d exposure periods at the tested environmentally relevant concentrations. Further studies on influence factors of uptake, elimination, and toxic molecular mechanisms should be explored for environmentally realistic MPs.

## Data Availability

Data is contained within the article.

## Supplementary Materials

The following supporting information can be downloaded at: https://www.mdpi.com/article/10.3390/toxics10020087/s1, Figure S1: Microbeads (A) extracted from facial scrub and those (B) used for the bioassays, Figure S2: Size distribution of microbeads used for bioassays, Figure S3: Fourier transform infrared (FT-IR) spectrum of the studied microbeads, Figure S4: Feces of adult (A) and young (B) *B. glabrata* in control groups over 48-h and 21-d exposure, Table S1: Overview of the experimental design for experiment I-II, Table S2: Statistical parameters of size distribution (μm) of microbeads in different media for 48-h bioassay, Table S3: Statistical parameters of size distribution (μm) of microbeads in different media for 21-day bioassay.
