# Supplementary material for: Uptake, Elimination and Effects of Cosmetic Microbeads on the Freshwater Gastropod Biomphalaria glabrata
Source: Toxics. 2022 Feb 14;10(2):87. doi: 10.3390/toxics10020087 (PMC8880069; doi:10.3390/toxics10020087)
Supplement: Supplementary file 1 [file toxics-10-00087-s001.zip › toxics-1567745-supplementary.pdf]

toxics

MDPI

Supplementary Materials: Uptake, Elimination and Effects of Cosmetic Microbeads on the Freshwater Gastropod *Biomphalaria glabrata*

Ying Wang, Alice Baynes, Kofi O. Renner, Mingxing Zhang, Mark D. Scrimshaw and Edwin J. Routledge

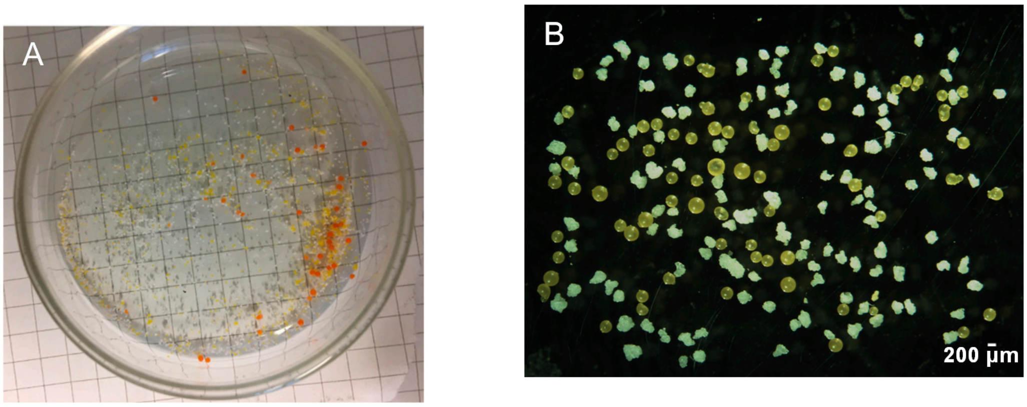

(A) (B)

Figure S1. Microbeads (**A**) extracted from facial scrub and those (**B**) used for bioassays.

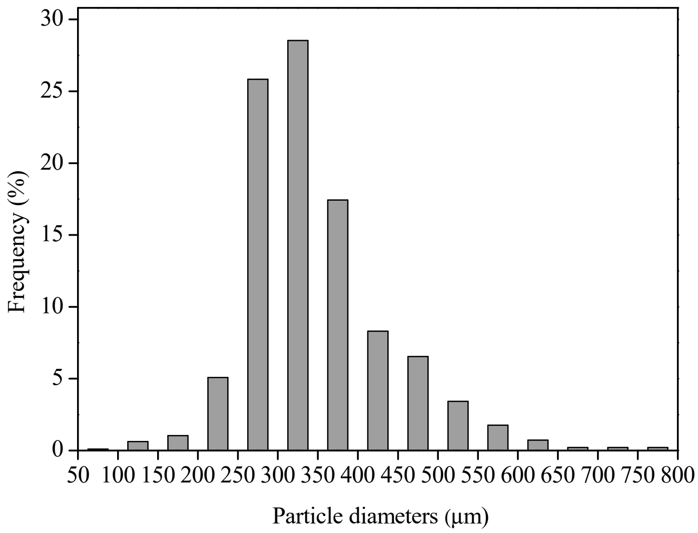

Figure S2. Size distribution of microbeads used for bioassays.

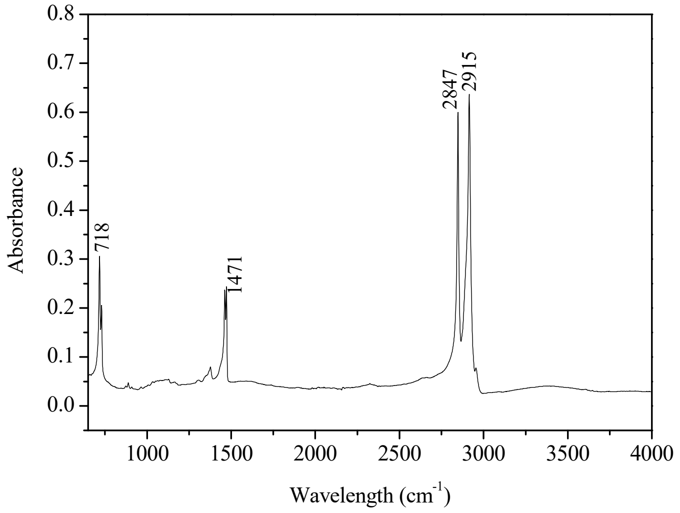

Figure S3. Fourier transform infrared (FT-IR) spectrum of the studied microbeads.

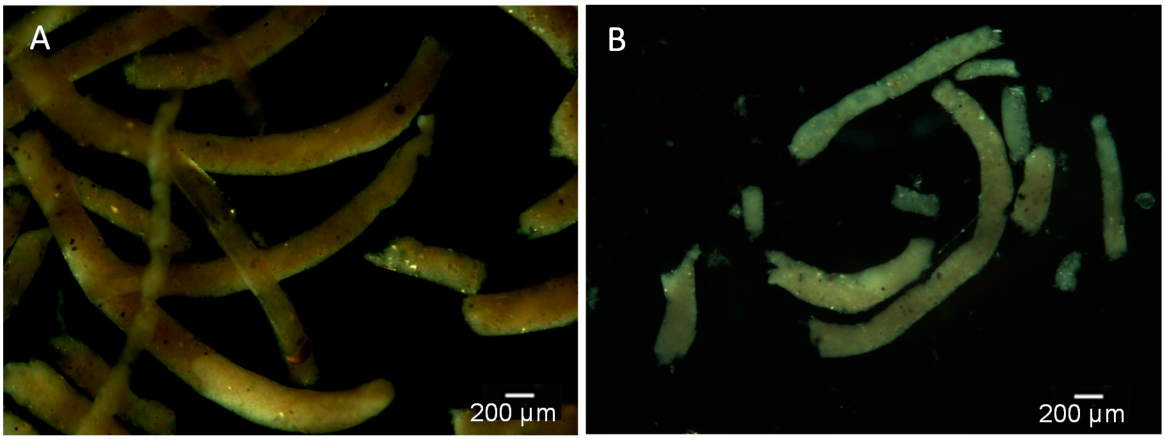

Figure S4. Faeces of adult (**A**) and young (**B**) *B. glabrata* in control groups over 48-h and 21-d exposures.

Table S1. Overview of the experimental design for experiment I-II.

**Experiment**		**Content**	**Endpoints or operation**	**Test concentrations**	**Number of animals /replicate/treatment**	**Replicates**	**Number of animals in total**
**I**	Short-term (48 h)	Ingestion and egestion (I(a))	Microplastic quantification	0, 4 particles/mL (5 mg/L)	5 (4 for Exp. I(a))	6	4 × 2 treatments ×6 replicates = 48
		Acute toxicity (I(b))	Growth, survival				5 × 2 treatments × 6 replicates = 60
II	Long-term (21 d)	Ingestion and egestion II(a)	Microplastic quantification	0, 0.4 particle/mL (0.5 mg/L)	5 (4 for Exp. II(a))	6	4 × 2 treatments × 6 replicates = 48
		Chronic toxicity II(b)	Growth, survival				5 × 2 treatments × 6 replicates = 60

Table S2. Statistical parameters of size distribution (μm) of microbeads in different media for 48-h bioassay.

	Fragment				Sphere			
	**Tissue**	**Faeces**	**Exposure medium**	**Standard**	**Tissue**	**Faeces**	**Exposure medium**	**Standard**
**Min**	195	141	155	171	128	111	86	83
**Max**	906	816	672	789	629	443	427	442
**Average**	415	384	386	371	332	288	288	291
**SD**	109	103	104	90	66	65	57	52
**Number**	755	1102	79	660	351	67	162	304

Table S3. Statistical parameters of size distribution (μm) of microbeads in different media for 21-day bioassay.

	Fragments			Spheres		
	**Tissue[a]**	**Faeces[b]**	**Standard**	**Tissue[a]**	**Faeces[b]**	**Standard**
**Min**	194	112	171	150	126	83
**Max**	900	842	789	558	636	442
**Average**	398	386	371	279	276	291
**SD**	115	91	90	62	72	52
**Number**	163	1106	660	375	86	304

[a] Tissue samples were collected at Day 21.

[b] Faeces samples were collected at Day 2, 4, 6, 8, 16 and 21; here shown is the statistical results for all of the faeces samples.
